# Accounting for behavioral responses during a flu epidemic using home television viewing

**DOI:** 10.1186/s12879-014-0691-0

**Published:** 2015-01-23

**Authors:** Michael Springborn, Gerardo Chowell, Matthew MacLachlan, Eli P Fenichel

**Affiliations:** Department of Environmental Science & Policy, University of California, 2104 Wickson Hall, One Shields Ave., Davis, CA 95616 USA; School of Public Health, Georgia State University, P.O. Box 3965, Atlanta, GA 30302-3965 USA; Division of International Epidemiology and Population Studies, Fogarty International Center, National Institutes of Health, 31 Center Dr, MSC 2220, Bethesda, MD 20892-2220 USA; Mathematical, Computational & Modeling Sciences Center, School of Human Evolution and Social Change, Arizona State University, 900 S. Cady Mall, Tempe, AZ 85287-2402 USA; Department of Agricultural & Resource Economics, University of California, 2116 Social Sciences & Humanities, One Shields Ave., Davis, CA 95616 USA; Yale School of Forestry and Environmental Studies, 195 Prospect St., New Haven, CT 06511 USA

**Keywords:** Epidemic model, Social distancing, A/H1N1, Influenza, SIR

## Abstract

**Background:**

Theory suggests that individual behavioral responses impact the spread of flu-like illnesses, but this has been difficult to empirically characterize. Social distancing is an important component of behavioral response, though analyses have been limited by a lack of behavioral data. Our objective is to use media data to characterize social distancing behavior in order to empirically inform explanatory and predictive epidemiological models.

**Methods:**

We use data on variation in home television viewing as a proxy for variation in time spent in the home and, by extension, contact. This behavioral proxy is imperfect but appealing since information on a rich and representative sample is collected using consistent techniques across time and most major cities. We study the April-May 2009 outbreak of A/H1N1 in Central Mexico and examine the dynamic behavioral response in aggregate and contrast the observed patterns of various demographic subgroups. We develop and calibrate a dynamic behavioral model of disease transmission informed by the proxy data on daily variation in contact rates and compare it to a standard (non-adaptive) model and a fixed effects model that crudely captures behavior.

**Results:**

We find that after a demonstrable initial behavioral response (consistent with social distancing) at the onset of the outbreak, there was attenuation in the response before the conclusion of the public health intervention. We find substantial differences in the behavioral response across age subgroups and socioeconomic levels. We also find that the dynamic behavioral and fixed effects transmission models better account for variation in new confirmed cases, generate more stable estimates of the baseline rate of transmission over time and predict the number of new cases over a short horizon with substantially less error.

**Conclusions:**

Results suggest that A/H1N1 had an innate transmission potential greater than previously thought but this was masked by behavioral responses. Observed differences in behavioral response across demographic groups indicate a potential benefit from targeting social distancing outreach efforts.

## Background

The series of flu-like outbreaks over the past decade illustrates the ongoing need for refinement of strategies to control and mitigate the impact of infectious diseases, including SARS in 2003 [1], the 2009 A/H1N1 (swine) influenza pandemic [2,3] and the emergence of a novel A/H7N9 (avian) influenza virus in 2013 [4]. In parallel to standard vaccination efforts, nonpharmaceutical interventions (NPIs) are a critical part of the management toolkit [5-7]. In particular, NPIs become even more relevant in the context of emerging infectious diseases when the availability of a vaccine may be substantially delayed. Chief among NPIs are strategies for enhancing social distancing, whether privately initiated or policy-directed (e.g., closing of schools, businesses and public events) [8]. While behavioral NPIs appear promising, it is important to evaluate empirically their efficacy since they can be costly [9] and could have unintended consequences, such as leading to a net increase in the long-run number of cases or increasing the total cost of the epidemic and policy response [10,11]. The potential for individual response to disease risk and policy presents a challenge for the measurement of the infectivity of a pathogen and design of policy directed social distancing [12,13]. Ferguson [14] argues that despite the need for a holistic approach, current models essentially ignore the feedback between epidemics and behavior.

Empirical analysis of the effect of social distancing behavior on epidemiological dynamics is of clear interest, but it has proven difficult to obtain representative data on actual behavioral responses to epidemics. Empirical investigation of the influence of behavior on flu-like transmission dynamics has been largely limited to binary proxies for behavior, specifically pre-scheduled [6,15] and epidemiologically driven [16] school closings and patterns of weekdays and weekends [17]. Though policy interventions are often coarse, individuals’ responses to policy and their own private decisions about risk are likely more nuanced [8]. Fenichel et al. [18] show that private risk reduction may have changed in subtle ways during the 2009 A/H1N1 epidemic. Caley et al. [19] estimate the change in infectious contact rates in Sydney, Australia from the 1918 influenza pandemic but do so indirectly by inferring changes in contacts based on the estimated reproduction number and proportion susceptible conditional on a given value for the reproduction number, R_0_.

We use novel data on variation in home television viewing behavior as a proxy for changes in the level of daily social interaction. We find a strong viewing behavior response in Central Mexico associated with the A/H1N1 influenza virus in April and May of 2009. The data reveal that proxy behavioral responses were greatest among children and wealthier socio-economic groups. Furthermore, we couple the behavioral response with an epidemiological model, and show that the A/H1N1 influenza virus was likely more transmissible than previously believed because the transmission potential was masked by behavioral responses.

To leverage the television viewing data for exploring the role of behavior during an epidemic, we extend the binary proxy for time varying infectivity in [20], where behavior can change at only one point in time, to allow for daily variation in behavior. Following [17], we decompose a standard model of the transmission rate into the two components of a contact rate and average transmission rate per contact. To inform changes in the contact rate, we use a daily proxy for changes in time spent by individuals in the home, namely variation in home television viewing. While viewing is an imperfect proxy for social distancing behavior, this data has several appealing attributes. The data are collected consistently prior to, during, and after epidemics in all major media markets worldwide. The sample is representative of the local population (by design) and can be disaggregated into various demographic subgroups. Typically the data is collected automatically and electronically (as in our sample) and do not rely on self-reporting. The viewing data in our application were obtained from IBOPE International net-AGB Nielsen Media Research, the largest private research and audience measurement firm in Latin America.

We contribute to the literature by examining variation in the behavioral response across time and demographic subgroups and by calibrating and analyzing dynamic behavioral disease transmission models. First, we quantify the dynamic nature of the behavioral response to the 2009 A/H1N1 influenza pandemic and public intervention in Central Mexico. We show that the aggregate response is not constant and describe how it varies systematically over time. Next, we unpack the aggregate dynamic into demographic subgroups and show how certain age groups and/or socio-economic groups respond more strongly than others. Turning to the modeling of disease transmission dynamics, we assess whether accounting for daily changes in contacts better accounts for the variation in new cases. We then explore the potential for bias in the standard model from ignoring underlying changes in behavior. Previous simulation analysis has shown that intervention focused on children is particularly effective in reducing the attack rate of influenza [21]. We examine how accounting for heterogeneity between adults and children alters conclusions. In the next section we first show how the basic transmission model can be extended to incorporate dynamic behavior and then describe the data and model estimation approach in detail.

## Methods

### Standard epidemiological model

We model the 2009 A/H1N1 epidemic in Central Mexico using an SEIR epidemiological model [22-24]. We define three different model formulations: one that does not account for any behavioral changes, one that assumes that behavioral change is constant throughout the government-imposed health interventions, and one that assumes that behavioral change can be estimated by daily television viewing data. For each model, individuals in the population, of size N, are classified by health status of individuals in into four states in each period, t: susceptible (S_t_), exposed (infected but not yet infectious), (E_t_), infectious (I_t_), and recovered (R_t_). The transition dynamics between health states are described by a system of difference equations:

1 $$ \begin{array}{c}{S}_{t+1}-{S}_t=-{\beta}_t{S}_t{I}_t/N\\ {}{E}_{t+1}-{E}_t={\beta}_t{S}_t{I}_t/N-\kappa {E}_t\\ {}{I}_{t+1}-{I}_t=\kappa {E}_t-\gamma {I}_t\\ {}{R}_{t+1}-{R}_t=\gamma {I}_t,\end{array} $$

where *β*_*t*_ is the transmission rate, κ is the rate at which incubating individuals progress from the exposed to the infectious health status (or the inverse of the latent period) and γ is the recovery rate (or the inverse of the recovery period).

In the standard (SD) model β_t_ is becomes a constant scalar. This confounds the combined effect of contacts and the probability of transmission from a contact [12]. In the classical transmission model, the behavior governing contacts is assumed to be fixed. Yet for many human diseases, including influenza, behavioral shifts and NPI likely play an important role in the transmission process.

### Behavioral epidemiological model

To generalize the classical model we decompose β_t_ into the likelihood of transmission conditional on a contact (ρ_0_) and the average number of contacts experienced by individuals $\left(\overline{\mathrm{C}}\right)$:

2 $$ {\beta}_t^{SD}={\rho}_0\overline{C}. $$

The parameters ρ_0_ and $\overline{\mathrm{C}}$ are not uniquely identified since they enter the model as a product. Nevertheless, ρ_0_ can be estimated following [17] and using population estimates from the literature for $\overline{C}$.^a^

Despite differentiating between the likelihood of transmission from a contact and the number of contacts, ${\beta}_t^{SD}$ is assumed to be constant. We explore two alternatives that relax the assumption of a constant transmission rate. The first extension to facilitate a time-varying transmission rate is to allow for two different, but otherwise constant, levels in *β*_*t*_ over time. Following [20], we model the behavioral response as a fixed effect (FE) (i.e. using a dummy variable) for the duration of a time period given by τ, for example during a particular public health intervention,

3 $$ {\beta}_t^{FE}=\left({\rho}_0+{\mathbf{1}}_{\tau }(t){\rho}_1\right)\overline{C}, $$

where ρ_0_ is a baseline marginal transmission rate (per contact), ρ_1_ is a shift in the marginal baseline transmission rate during the window τ, and 1_τ_(t) is the indicator function, equal to one when t ∈ τ, and zero otherwise.

Second, we propose a flexible response model that allows for daily variation in behavior. Given the availability of an empirical proxy for changes in contact rates, we relax the assumption of fixed contact rates. Let Δ_t_ represent the percentage deviation from the average $\overline{\left(\mathrm{C}\right)}$ for a given period t. A dynamic behavioral (DB) transmission function that is similar in form the Equations (2) and (3) but accounts for variation in the contact rate is:

4 $$ {\beta}_t^{DB}=\left({\rho}_0+{\rho}_1{\Delta}_t\right)\overline{C}. $$

Relative to the SD model in Equation (2), the DB transmission rate model includes an additional term $\left({\rho}_1{\Delta}_t\overline{C}\right)$ capturing an additive effect of any behavioral response. The SD model (2) is nested within both the FE model (3) and the DB model (4): ${\beta}_t^{SD}={\beta}_t^{FE}\left({\rho}_1=0\right)={\beta}_t^{DB}\left({\rho}_1=0\right)$. Under all three models, the subset of the population N in each of the health states changes over time. The only other potentially dynamic component is the transmission rate *β*_*t*_, which is either fixed (SD model), takes one of two constant values over time (FE model), or varies daily (DB model).

### Epidemiological data

To examine the implications of social distancing we focus on the initial outbreak of A/H1N1 in Central Mexico, in the spring of 2009.^b^ We obtained laboratory confirmed pandemic A/H1N1 influenza cases from April 1 to May 20 in Central Mexico from a prospective epidemiological surveillance system that was established in response to the 2009 influenza pandemic by the Mexican Institute for Social Security (IMSS) [25]. These data are presented in Table 3 in Appendix A. IMSS is a tripartite Mexican health system that relies on a network of over 1,000 primary health-care units and 259 hospitals nationwide, and covers ~40% of the Mexican population. Importantly, testing rates for novel A/H1N1 influenza remained stable at ~33% [20]. Chowell et al. [20] show that the age distribution of the population affiliated with IMSS is generally representative of the general population of Mexico, rejecting the hypothesis that the distributions are significantly different. Furthermore they note that the male-to-female ratio among the population affiliated with IMSS (47:53) is similar to that of the general population (49:51).

On April 15th 2009, the Mexico Ministry of Health began receiving informal indications of a severe pneumonia in metropolitan Mexico City [3,26]. The novel influenza A/H1N1 virus was confirmed by U.S. and Canadian labs for multiple Mexican patients from April 22–24. On Friday, April 24^th^, the federal government announced the closure of public schools for metropolitan Mexico City, and a public awareness campaign was initiated by the Ministry of Health. Further “social distancing measures” involved closing restaurants and entertainment venues and cancelling large public events [26]. After May 9, the infection rate declined dramatically and large public health interventions were lifted [20]. Students resumed school on Monday, May 11. The window *τ* = {April 24, …, May 10} is used in the FE model for the sub-period over which we might expect to observe an effect due to social distancing. We also considered alternative dates for the start of this window, from April 10^th^ through April 23rd, but none were statistically preferred as explained further in the results. A graphical timeline of events related to the outbreak is provided by Chowell et al. [20] (Table 1).

**Table 1:** **Summary statistics for daily percentage deviation from the long-run mean ATV** (*Δ*_*t*_) **for various demographic groups**

		**Statistics for Δ**_**t**_ **within the intervention period (τ)**			
**Group**		**Range**	**Mean**	**Mean = 0 (p-value)**	**Equal means within class (p - value)**
Aggregate		[−1.4%, 22.6%]	13.6%	<0.001	.
Age class	Children	[−4.7%, 46.2%]	23.7%	<0.001	0.001
	Adults	[−6.5%, 21.8%]	8.9%	<0.001	
SEL class	Low	[−0.2%, 32.0%]	17.8%	<0.001	Low-med: 0.23
	Medium	[0.4%, 32.1%]	15.3%	<0.001	Med-high: 0.51
	High	[−3.0%, 21.6%]	11.8%	<0.001	Low-high: 0.04
Time of day	Daytime	[−3.7%, 30.7%]	18.4%	<0.001	0.005
	Nighttime	[−4.1%, 17.0%]	9.6%	<0.001	

Ethics Committee approval was not necessary according to local regulations. All the data were de-identified. Data employed in this study are routinely collected for epidemiological surveillance purposes.

### Behavioral data

We use data on home television viewing in metropolitan Mexico City as a proxy measure for dynamic behavioral response in Central Mexico during the influenza outbreak. The logic of this approach relies on two key assumptions. First, we assume that time spent watching television increases in time spent in the home, and that a linear approximation is sufficient to capture this behavior.^c^ With respect to an individual’s daily time allocation, since we are mainly concerned with time spent at home or not at home, an increase in the former subtracts from the latter. Second, we assume that the number of contacts an individual makes is proportional to the time spent outside the home.

Viewership data for Mexico City were obtained from IBOPE International net-AGB Nielsen Media Research, the largest private research and audience measurement firm in Latin America.^d^ The specific measure used was individual daily average time viewed (ATV), which is given by the aggregate number of hours viewed by everyone in the sample divided by the number of individuals in the sample (including those with no viewing in a given period). The data reflect aggregate observations for individuals (not households) in a given demographic group. IBOPE’s sample is composed of an ongoing panel of individuals, balanced across demographic characteristics to be representative of the population of Mexico City. Daily data were obtained for the months of April and May in 2009. With respect to data on daily confirmed cases of influenza and average TV viewership, ethics committee review was not relevant since all data were de-identified, aggregated before acquisition and collected under existing conditions (i.e. there were no experimental treatments). Similarly, since the data were gathered through existing mechanisms and not for our study, obtaining written informed consent from participants was not relevant.

We used the percentage deviation in average television viewership (relative to the non-intervention period) as a proxy for the percentage deviation in contacts. We choose this simple form for the proxy since a parameterized model of raw contacts as a function of television viewing is not available. Let $\overline{ATV}$ represent the baseline (non-intervention period) mean of ATV_t_ over an extended time horizon from both before and after the public response to the outbreak, but not during. The baseline period used to determine $\overline{ATV}$ is April 1-April 23 and May 10-May 31, which includes April and May of 2009, excluding the period τ. $\overline{ATV}$ for our sample is 1.7 hours per day (with a minimum and maximum ATV_t_ over the baseline period of (1.5, 1.9)). The time-varying deviation from the baseline mean ATV_t_ is given by ${\varDelta}_t=\left(AT{V}_t-\overline{ATV}\right)/\overline{ATV}$.

We considered both a single homogenous population and a heterogeneous population divided into two groups: adults (age 18 and above, denoted A) and children (individuals below the age of 18, denoted K). For the heterogeneous population model, the disaggregated viewership data allowed for inference on how the behavior of adults and children varied over time. The extension of the homogenous population transmission model in (1) to the heterogeneous subgroup setting is presented in Appendix B. Information is not available to characterize how changes in contacts made by one group (e.g. adults) might differ between contacts they make within the same group (e.g. adult-adult contact) versus another group (e.g. adult-child contact). Therefore, we make the simplifying assumption that deviation in the contact rate for a member of group *i* is uniform across the different groups they may come in contact with; we used a single time series to inform deviations in children’s contacts with either adults or children (*Δ*_*t*, *K* → *A*_ = *Δ*_*t*, *K* → *K*_ = *Δ*_*t*,*K*_) and another single time series similarly for adults (*Δ*_*t*,*A* → *K*_ = *Δ*_*t*, *A* → *A*_ = *Δ*_*t*,*A*_).

We modeled the age-specific contact rates for school-age children and adults for central Mexico based on survey contact data collected from several European countries [27]:

5 $$ \mathbf{C}=\left[\begin{array}{cc}\hfill {\overline{C}}_{K\to K}\hfill & \hfill {\overline{C}}_{K\to A}\hfill \\ {}\hfill {\overline{C}}_{A\to K}\hfill & \hfill {\overline{C}}_{A\to A}\hfill \end{array}\right]=\left[\begin{array}{cc}\hfill 8.9\hfill & \hfill 5.5\hfill \\ {}\hfill 1.9\hfill & \hfill 9.3\hfill \end{array}\right]. $$

The average contact rate for the homogenously mixing population, $\overline{C}=6.1$, is given by the population-weighted average of **C**.

### Model estimation

We set the population of Central Mexico to N = 5.3*10^7^ individuals [28] and follow [17] in setting the mean probability of an infection being laboratory-confirmed A/H1N1 influenza at $\overline{\upvarphi}=0.0015$. This estimate of is constructed as the product of the symptomatic rate (65% [29,30]), the hospitalization rate (0.45% [31]), and the probability of an infected, hospitalized individual being identified as having A/H1N1 (50%). We control for observed variation in the rate that hospitalized cases were tested by scaling the mean probability of confirmation by the observed deviation from the mean testing rate: ${\phi}_{\mathrm{t}}=\overline{\upvarphi}\left({\mathrm{TR}}_{\mathrm{t}}/\overline{\mathrm{TR}}\right)$. Testing rate data were obtained from IMSS (the same source as described above for the case data). We set the fraction initially infected on day 1 of the time period (April 1) at π = 1.9 × 10^− 5^, such that given the population and the probability of confirmation, one case is confirmed on the first day. Consistent with [5,32,33], the daily rate of progression from latent to infected health status and the recovery rate are set to κ = 0.67 and γ = 0.5, respectively.

The main coefficients of interest for estimation are the parameters of the transmission rate functions for each of the three models. Let ρ represent the vector of marginal transmission rate parameters, given by the scalar [ρ_0_] for the SD model and the vector [ρ_0_, ρ_1_] for the FE and DB models. Model parameters were estimated by maximum likelihood. We assumed that the observed number of confirmed new infections each day, ${I}_t^c$, follows a Poisson process with a mean arrival rate *λ*_*t*_(*ρ*) given by the number of new observed infections predicted by the disease model, *ϕ*_*t*_*κE*_*t*_. The log-likelihood function is:

6 $$ L={\displaystyle \sum_{t=1}^T\left({I}_t^c \ln \left[{\lambda}_t\left(\rho \right)\right]-{\lambda}_t\left(\rho \right)- ln\left({I}_t^c!\right)\right)}. $$

Development of the log-likelihood function is explained in further detail in Appendix C.

Because maximum likelihood estimates can be sensitive to the choice of initial values provided to the numerical optimization algorithm, we used a multiple starting point solver in Matlab (version R2013a) designed to identify the global optimum. For each model, the solver was run for each of M different randomly drawn starting vectors for the unknown parameters in ρ. We set M equal to 50 for the standard model (one parameter) and 100 for the alternative models (two parameters). From this set of local maxima, the solution with the greatest likelihood was selected as the estimate for the global maximum. We estimated 95% confidence intervals for the parameters using the likelihood ratio method [34]. To test for statistically significant differences in performance, when comparing the SD model against the FE and DB models we used a likelihood ratio test, since the SD model is nested within both of the alternatives (FE and DB). Since the FE and DB models are not nested, the standard likelihood ratio test is not feasible. Following [35], we used a Cox non-nested test with a parametric bootstrap (see Appendix D for details).

## Results and discussion

### Dynamic behavioral response

**Figure 1:**
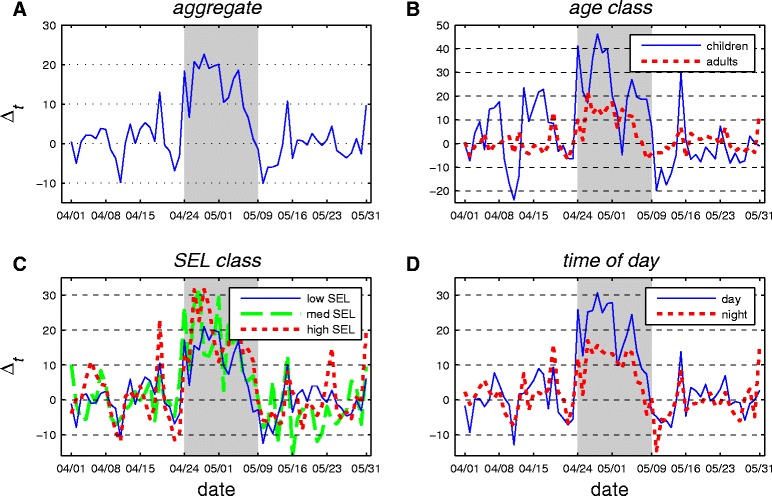
**Percentage deviations from mean daily individual average time viewed (Δ**_**t**_ **) for various demographic groups in Mexico City.** Panel **A** shows deviations for the aggregate population. Panel **B** distinguishes between adults and children. Panel **C** differentiates by socioeconomic level (SEL). Panel **D** presents the daytime versus nighttime response. The shaded area in each graph represents the intervention period τ.

In Figure 1 we present the dynamic behavioral response time series for *Δ*_*t*_ (percentage deviation from mean ATV) in Mexico City during April and May 2009 in aggregate (Figure 1A) and for various demographic and time subgroups (Figure 1B-D). The range and mean for this variable over the limited intervention period (τ) is presented in Table 1. A positive deviation (*Δ*_*t*_ > 0) indicates that an above average amount of time was spent in home TV viewing and, by inference, in the home. The mean level of *Δ*_*t*_ over the period τ is positive and, as shown by a one-sample *t*-test, significantly different from zero at the 1% level for the aggregate population and each subgroup considered here (see Table 1).

The dynamic path of *Δ*_*t*_ for the aggregate population is presented in Figure 1A. Outside of the shaded intervention window (τ), this measure has a mean of zero (by construction) and typically falls within a range of +/− 5%. During the period τ, *Δ*_*t*_ shifts demonstrably upwards. This behavioral response is strongest in the first week (approximately 20%) before gradually tapering off to near zero by the end of the intervention period. This pattern suggests that the population’s capacity for social distancing might be limited in duration; before the public health intervention concluded, there was a substantial decline in the behavioral response relative to the peak in the first week. (Alternatively, it may be that the level of viewing per unit of time spent in the home fell as individuals switched to other in-home activities.) After the NPI concluded there was a period of reduced viewing activity in the home (*Δ*_*t*_ < 0). Specifically, *Δ*_*t*_ reached its most negative point on May 10^th^ at −10.5%. Outside of the post-intervention dip, *Δ*_*t*_ dropped below −10% on only one other day. As further evidence that the dip was likely not a coincident random event, we find that this dip persisted at 5% below the non-NPI period mean for four consecutive days—there are no other instances in the data when *Δ*_*t*_ falls below 95% of the mean for more than a single day. While the causal mechanism behind these dynamics is not known with certainty, one possibility is that this multi-day period of suppressed in-home activity compensated for forgone social and commercial activities from earlier in the intervention period. The observation of reallocation of risky activities in time is common in the public health literature. Following the introduction of antiretroviral treatment for HIV/AIDS [28,36] find empirical evidence of increased sexual risk taking. Boyes and Faith [2] show that when alcohol consumption is banned at college football games that total alcohol consumption may rise through substitution effects in periods sandwiching the game. Finally, Graff Zivin and Neidell [37] find that while Southern California residents curtail outdoor activity on days with poor air quality, if the episode is prolonged the behavioral response dissipates rapidly.

The age class breakdown for *Δ*_*t*_ presented in Figure 1B shows a substantial difference in response between children and adult subgroups during the intervention period. The mean (23.7%) and the maximum (46.2%) behavioral response of children is more than twice as large as the response observed for adults (see Table 1). The difference in responses is statistically significant at the 1% level as indicated by a two sample *t*-test.

The data from IBOPE are disaggregated into three socioeconomic levels (SELs) based on a set of household characteristics, including the size and amenities of the home, appliance ownership, automobile ownership, and level of education (Figure 1C). During the intervention period, on average the high SEL group shows a response that is over 50% greater than that of the low SEL group. This difference is significant at the 5% level. The medium SEL class displays an intermediate response (Table 1).

Finally, we consider variation in the response by time of day, specifically daytime (6 am-6 pm) versus nighttime (6 pm-6 am) (Figure 1D). The mean daytime response is approximately twice as strong as the nighttime response (Table 1). This is not surprising given that time spent in the home is lower during the daytime to begin with and thus presents a larger opportunity for adjustment.

The time path for each of the subgroups discussed above follows a path that is qualitatively similar to that of the aggregate population, showing a strong initial positive response that largely or entirely decays before the end of the intervention. For each subgroup comparison considered here, there was a significant difference in the average level of the behavioral response.

### Transmission model estimation

**Figure 2:**
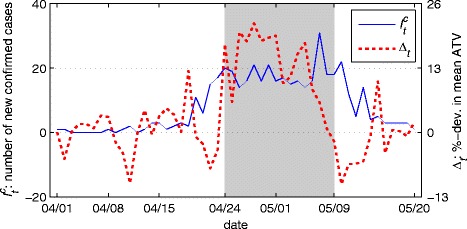
**Time series for the number of new confirmed cases (** ${\mathbf{I}}_{\mathbf{t}}^{\mathbf{c}}$ **, left axis) and percentage deviation from mean daily individual average time viewed (** *Δ*_***t***_ **, right axis) for 50 days beginning April 1, 2009.** The shaded area in each graph represents the intervention period τ.

The maximum likelihood parameter estimates for each model are based on T = 41 days of observations, stretching from April 1 through the end of the intervention period on May 11 (Table 2). Figures illustrating the log-likelihood profile for each model are presented in Appendix E. The time frame used corresponds to the period of time considered in [20]. After this period, additional cases attenuate substantially as shown in the time series of ${I}_t^c$ (Figure 2). We focus on this initial 41 day period since the performance of each model (in terms of log-likelihood values and residuals) becomes increasingly poor as more of the post-intervention period is included

**Table 2:** **Maximum likelihood parameter estimates**

		**Standard (SD)**	**Fixed effect (FE)**	**Dyn. behav. (DB)**
Transmission parameters	ρ_0_	0.0565	0.0642	0.0647
		(0.0561, 0.0568)	(0.0640, 0.0644)	(0.0644, 0.648)
	ρ_1_		−0.0233	−0.1516
			(−0.0257,-0.0208)	(−0.1519, −0.1513)
Observations		41	41	41

95% confidence intervals are in parentheses.

.

The degree to which accounting for changes in contacts better accounts for the variation in new cases is one of our core research questions. Results show that the standard model is indeed incomplete—we reject the SD model in favor of both the DB model (p < 0.01) and FE model (p < 0.01). However, we do not find that the DB model outperforms the FE model. In fact we reject the DB model in favor of the FE model (p < 0.01). To see why it might be the case that a simple fixed effect is preferred in this case to the dynamic, data-driven behavioral model, consider the time series for ${I}_t^c$ and *Δ*_*t*_ presented in Figure 2. Consistent with expectations under the DB model, when the social distancing proxy *Δ*_*t*_ begins to surge on April 24^th^ (day 24) the number of new confirmed cases plateaus. However, when *Δ*_*t*_ declines in early May while infections are still common, the number of new confirmed cases $\left({I}_t^c\right)$ does not grow in a sustained fashion but rather, after a slight delay, begins to fall. Thus the dynamics of initial and early intervention period of the outbreak are consistent with the DB model but the late intervention period is not.

**Figure 3:**
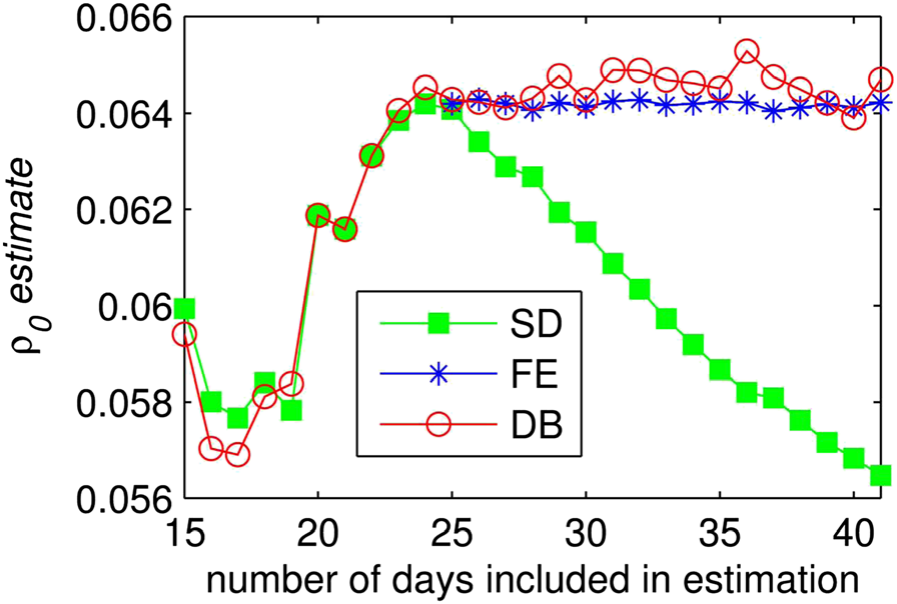
**Estimates for the baseline rate of transmission for the three models: standard (SD), fixed effect (FE) and dynamic behavioral (DB).**

Given that both the FE and DB models outperform the SD model, we explored the potential for biased estimates of the transmission parameter in the SD model as a potential shortcoming of ignoring behavioral change. Estimates of the baseline transmission rate (ρ_0_) in Table 2 show that while the DB and FE models are in essential agreement, the SD estimate is 12% lower. To explore whether this difference is idiosyncratic or systematic we re-estimate each of the three models starting with only the first M days of data for M ∈ [15, 41]. We exclude the FE model for M ∈ [15, 24] since this model is not differentiated from the SD model until the intervention begins on April 24^th^. In Figure 3 we present the resulting estimates of ρ_0_. We find that estimates are variable but roughly consistent across models through April 24^th^. This is not surprising given that before the public health intervention began on April 24^th^ our proxy suggests that behavior had yet to shift discernibly. After this point, estimates of ρ_0_ for the DB and FE models remain roughly stable near 0.064 while the baseline transmission coefficient for the SD model declines monotonically. Thus over the intervention period when behavioral response is strong, the SD estimate of ρ_0_ falls each day to account for the new factor. In contrast, models that allow for a behavioral shift result in estimates for baseline transmission that are essentially level over time.

As a practical matter, this bias in the SD model has important implications for public health and forecast error. First, the SD model provides an estimate of ρ_0_ substantially lower than models with behavior. This suggests that A/H1N1 virus is more infectious, but this infectiousness is masked by behavioral shifts. Second, the SD model results in substantial forecast error, a result shown using simulation in [13] to emerge when human adaptive behavior is important in epidemiological systems.

### Forecasting error comparison

**Figure 4:**
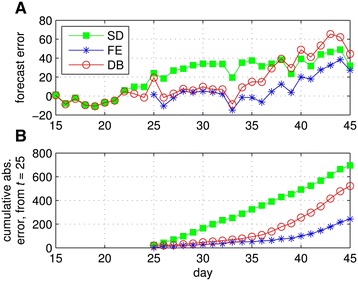
**Error from forecasting new confirmed cases over a four-day horizon conditional on the number of days observed under the standard (SD), fixed effect (FE) and dynamic behavioral (DB) models.** Panel **A** shows daily forecast error and Panel **B** shows cumulative absolute error starting from day 25.

In Figure 4 we present forecast error over a four-day horizon for time series of increasing length from M ∈ [15, 41]. The exercise is meant to capture the public health official’s problem of estimating the current state of an outbreak based on observed cases to date. We assume that there is a four-day lag between the date of testing and reporting of all confirmed cases, a typical lag for reporting infectious disease outbreaks. Thus forecast error appearing in the figure for day M = 15 represents error made on day 19 conditional on case data that is complete through day 15. We assume that behavioral data (*Δ*_*t*_) is available across this four day lag. From the raw forecast error in Figure 4A, it is clear that prediction performance for the SD model becomes poor relative to the alternatives shortly after the intervention on day 24. From this point on, the SD model leads to systematic over-prediction of the number of new cases. DB model performance deteriorates next towards the end of the intervention period. Finally, by the time the intervention concludes, all three models systematically over-predict new cases. This suggests that factors absent from the models considered here are important for capturing post intervention dynamics (e.g. personal protective measures to reduce risks per contact).

We estimated the transmission model results above assuming a single homogenous population. However, differences in the behavioral response (*Δ*_*t*_) for children versus adults presented above motivate exploration of age-class heterogeneity. When we modeled children and adults as separate populations (with separate time series for *Δ*_*t*_ in the behavioral model), but constrained transmission parameters to be the same for both populations, estimates were not significantly changed. We further tested an extended model in which transmission parameters (*ρ*_0_, *ρ*_1_) were free to vary between the two groups. This model was not statistically significantly different for either the SD (p = 0.31), DB model (p = 0.41), or FE model (p = 0.12) at the 10% level. For this FE model, relative to the homogeneous (baseline) case, the coefficients ρ_0_ and ρ_1_ were roughly 50% larger in magnitude for children and 90% smaller in magnitude for adults. This evidence is not conclusive, but hints that infections between children and from children to adults might have been a leading driver of disease dynamics—and also most sensitive to intervention. However, this effect is too small and imprecisely estimated to assert with statistical significance.

While we failed to find a significant difference in the transmission coefficients between children and adults, this does not mean that there were not significant differences in these populations. Recall that we controlled for differences between children and adults in the baseline contact rate as specified in the matrix **C**. When this matrix was replaced with the average $\left(\overline{C}\right)$ a significant difference emerged between the homogeneous and heterogeneous coefficient specification for both the SD (p < 0.01) and DB models (p = 0.08) but not for the FE model.

### Sensitivity analysis

We examined the sensitivity of transmission model results to several alternative assumptions. First, given the temporal mismatch between the case and behavioral time series in Figure 2, we explored whether the relative preference for the FE model continued to hold under extensions in the latent period, i.e. the number of days individuals were infected but not infectious. In the baseline model the latent period was set to 1/κ = 1.5. The performance of the DB model relative to the FE model was robust to alternative assumptions on the latent period, including 2, 3 or 4 days. We also considered whether idiosyncratic variation or “noise” in the ATV variable might hinder the DB model. As a simple test we set a +/−5% threshold for the Δ_t_ measure—any variation that did not exceed this band was set to zero. This did not qualitatively change results. Qualitative results were also not sensitive to a nonlinear quadratic form for the DB model.

Convergence in the performance of the DB and FE models was found when the number of days included in the estimation was limited. For all time series that included 38 days or less, we failed to reject one model in favor of the other. However, after this time frame the FE model emerges as the preferred model (e.g. p < 0.01 at 39 days).

For the FE model, we also considered alternative dates for the start of the intervention window, from April 10^th^ through our baseline window start date of April 24^th^. For each of these alternative specifications we found that the associated parameter ρ_1_ was statistically significantly different from zero. However, we also found that the log-likelihood was greatest for the FE window beginning on April 24^th^ (our baseline specification) illustrating that none of the alternative start dates was statistically preferred.

The final parameter examined in our sensitivity analysis was the mean probability of confirmation. Our baseline level for $\overline{\upvarphi}$ implies that 1.2% of the population was infected by the end of the spring wave (conditional on the observed number of cases and total population). We examined sensitivity to an alternative scenario in which 10% of the population contracts the disease, which implied a mean probability of confirmation of $\overline{\upvarphi}=8.1\times {10}^{-5}$. Results from this alternative low probability of confirmation scenario were not qualitatively different.

### Counterfactual behavioral response

**Figure 5:**
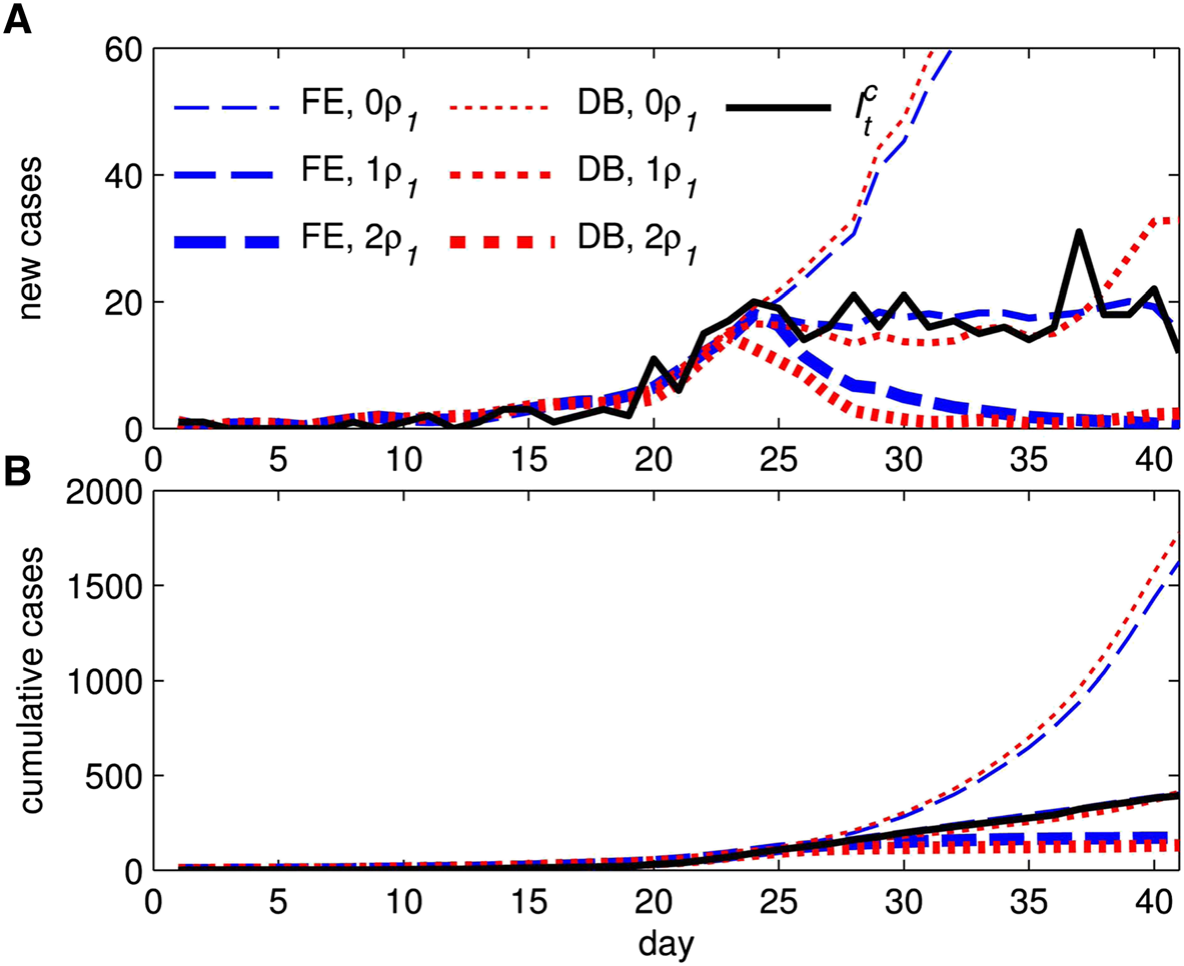
**Fitted daily cases (A) and cumulative cases (B) for the unaltered fixed effect (FE) and dynamic behavioral (DB) models (1ρ**_**0**_ **) and two alternatives where the behavioral response is either eliminated (0ρ**_**0**_ **) or doubled (2ρ**_**0**_ **).** Observed newly confirmed cases $\left({I}_t^c\right)$ are also provided for comparison.

We explore two alternative scenarios in which the behavioral response to the epidemic is either non-existent or enhanced. We present the path of new confirmed cases under these alternatives, along with fitted curves from the baseline models in Figure 5A. Under the first alternative, to eliminate the behavioral response, we multiply the ρ_0_ term by zero (0ρ_0_, thin lines). Under the second alternative, to enhance the behavioral response, we multiply the ρ_0_ term by two (2ρ_0_, thick lines). Fitted curves from the unaltered baseline models (1ρ_0_, medium lines) and ${I}_t^c$ are provided for comparison. For the baseline models, the fit of the DB and FE models is similar until the final few periods in which the DB fit diverges from the observed path $\left({I}_t^c\right)$. The importance of the behavioral response is evident. With no behavioral response, the projected path of new cases increased sharply, more than quadrupling (Figure 5B) for both models by day 41. Alternatively, with a doubling of response, attenuation of new cases occurs approximately two weeks earlier and cumulative cases by day 41 are cut in half.

## Conclusion

We used novel data on variation in home television viewing behavior as a proxy for changes in the level of daily social interaction in Central Mexico during the 2009 A/H1N1 influenza pandemic. Results from both behavioral models (FE and DB) suggested that social distancing was a key factor in constraining the initial wave of A/H1N1 in Central Mexico. In the absence of a behavioral response, the estimated counterfactual path of new cases escalated rapidly in initial weeks rather than stabilizing and eventually falling as was observed. The assumption of fixed behavior in the standard (SD) model led to shortcomings in estimation and prediction. Estimates of the baseline rate of transmission systematically shifted over time. If the baseline rate of transmission is interpreted as a measure of biological infectivity in the standard model, this is likely to lead to an underestimate of this parameter, as in our setting, given confounding effects of behavioral responses. This suggests that A/H1N1 had an innate transmission potential much greater than previously thought but this was masked by behavioral responses. This has implications for management advice including the allocation of resources between pharmaceutical and nonpharmaceutical interventions. Furthermore, the error in near term predictions of new cases through time was also substantially greater under the standard model compared to the behavioral models. This error was also systematic—the standard model consistently led to over-prediction in the number of new cases.

Comparing the behavioral models, we found that that the dynamic behavioral model was not preferred to the simpler fixed effect model. One explanation may be the imperfect nature of variation in viewership as a proxy for changes in public contact rate. For example, it is possible that during the public health intervention the observed increase in ATV_t_ was due to a greater share of home time allocated to TV viewing, rather than an increase in time spent at home. Or it could be the case that viewing per unit of time spent at home may be declining in time spent at home. Another explanation might be the inability at this time to empirically capture changes in behavior outside the home to reduce contacts or transmission (e.g. washing hands, wearing facemasks, and avoidance of coughing into open air). Bell [5] notes that while policies promoting social distancing may be effective against pandemic influenza, other individual behavioral measures should be either routine (e.g. hand and respiratory hygiene and disinfection of contaminated household surfaces) or considered for certain settings and risk levels (e.g. mask use).

We found that the home viewership response was stronger in the high (versus low) socioeconomic level (SEL) subgroup. This finding is suggestive but should be interpreted with care. On the one hand, individuals in the high SEL subgroup are arguably less constrained in adjusting contacts than those in the low SEL subgroup. For example, Kumar et al. [38] suggested that workplace policies can impinge on distancing measures and such workplace policies may be more binding on lower SELs. If this hypothesis were tested and verified, it would suggest the potential for targeting of social distancing polices to facilitate self-protective measures for low SEL individuals. On the other hand, it may be that the difference in response is an artifact of the behavioral proxy which might emerge, for example, if the relationship between home viewership and time spent at home differed systematically between SEL subgroups (e.g., if high SEL individuals respond more strongly because ownership of more televisions provides more opportunities to view).

In addition to varied responses across groups, we also found differences over time, namely attenuation in the behavioral response before the conclusion of the public health intervention. Furthermore, we found evidence of a rebound effect in which, after a prolonged period of elevated in-home activity there appeared to be period of suppressed activity. This is consistent with the historical analysis of Caley et al. [19] who found that as the perceived risk of the 1918 swine flu decreased in Australia, the public appeared to revert to normal behavior. Similarly, Fenichel et al. [18] found that air travelers’ adaptive to A/H1N1 dissipated after an initially strong response. Further studies of the 2009 A/H1N1 influenza pandemic in other regions with similar intervention measures (e.g. Hong Kong, [39]) could help to confirm and generalize the insights gleaned here.

While the dynamic behavioral model based on the home viewership proxy did not out-perform the simple fixed effect model, the results represent progress in identifying and unpacking the drivers behind this fixed effect. Going forward, further detailed data on private and public behavior during outbreaks would serve to identify behavioral effects on transmission with greater precision. For example, we did not model the effect of antiviral treatment. Capturing additional behavioral adjustments made outside of the home to reduce effective contacts is likely be important for explicit modeling of the behavior underlying disease transmission. To this end, there is value in allocating resources during an outbreak to consistently gather data on public and private protective actions, such as antiviral use or the use of face masks. Although transitioning from empirical analysis based on fixed effect measures of behavior to fully dynamic responses at finer time scales will require additional investment in data collection, potential benefits include the promise of informing more finely tuned and less costly public health interventions.

## Figures and Tables

**Figure 6:**
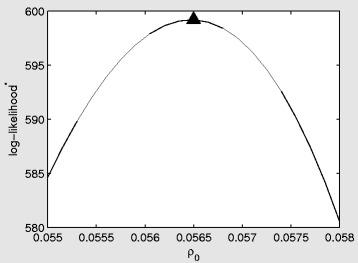
**Log-likelihood profile for the standard (SD) model as a function of the marginal transmission rate (per contact) ρ**_**0**_ **.** The triangle represents the maximum likelihood estimate from Table 2. The star on log-likelihood indicates that the constant term from the log-likelihood has been excluded.

**Figure 7:**
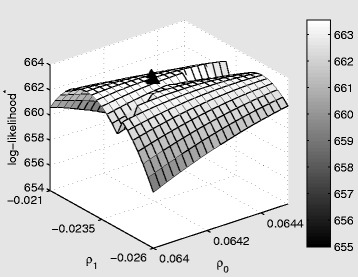
**Log-likelihood profile for the fixed effect (FE) model as a function of the marginal transmission rate (per contact) ρ**_**0**_ **, and the shift in the marginal baseline transmission rate during the intervention window, ρ**_**1**_ **.** The triangle represents the maximum likelihood estimates from Table 2. The star on log-likelihood indicates that the constant term from the log-likelihood has been excluded.

**Figure 8:**
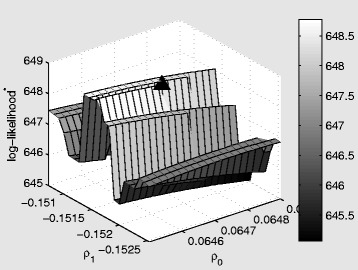
**Log-likelihood profile for the dynamic behavioral (DB) model as a function of the marginal transmission rate (per contact) ρ**_**0**_ **, and the behavioral response, ρ**_**1**_ **.** The triangle represents the maximum likelihood estimates from Table 2. The star on log-likelihood indicates that the constant term from the log-likelihood has been excluded.

## Appendix

A. Epidemiological data
  **Table 3:** **Laboratory confirmed pandemic A/H1N1 influenza cases from April 1 to May 20, 2009, in Central Mexico for children (under 18) and adults (18 and over)**
  	**Age**				
  **Date**	**Under 18**	**18 and over**			
  1-Apr-09	0	1	26-Apr-09	3	11
  2-Apr-09	0	1	27-Apr-09	9	7
  3-Apr-09	0	0	28-Apr-09	12	9
  4-Apr-09	0	0	29-Apr-09	7	9
  5-Apr-09	0	0	30-Apr-09	8	13
  6-Apr-09	0	0	1-May-09	7	9
  7-Apr-09	0	0	2-May-09	6	11
  8-Apr-09	0	1	3-May-09	8	7
  9-Apr-09	0	0	4-May-09	8	8
  10-Apr-09	1	0	5-May-09	10	4
  11-Apr-09	1	1	6-May-09	7	9
  12-Apr-09	0	0	7-May-09	19	12
  13-Apr-09	0	1	8-May-09	8	10
  14-Apr-09	1	2	9-May-09	11	7
  15-Apr-09	1	2	10-May-09	10	12
  16-Apr-09	0	1	11-May-09	7	5
  17-Apr-09	0	2	12-May-09	1	4
  18-Apr-09	1	2	13-May-09	9	5
  19-Apr-09	1	1	14-May-09	3	1
  20-Apr-09	2	9	15-May-09	2	3
  21-Apr-09	1	5	16-May-09	0	3
  22-Apr-09	4	11	17-May-09	1	2
  23-Apr-09	4	13	18-May-09	2	1
  24-Apr-09	6	14	19-May-09	2	1
  25-Apr-09	7	12	20-May-09	0	1
B. Multiple age class transmission model

In Table 3 we present the number of laboratory confirmed pandemic A/H1N1 influenza cases for each day in the period of study in Central Mexico from a surveillance system established in response to the 2009 influenza pandemic by the Mexican Institute for Social Security (IMSS) [25].

All three base models (SD, FE and DB) can be generalized to allow for age structure within the population. Social interactions may vary across demographic groups, for example children attending school versus working adults. We follow [17] in generalizing the system of differential equations for a homogenously mixing population in (1) to allow for variation in the transmission rate between demographic groups in the set G. Dynamics for each subgroup *i* ∈ *G* are given by:

7 $$ \begin{array}{c}{S}_{i,t+1}-{S}_{i,t}=-{S}_{i,t}{\displaystyle \sum_g^G{\beta}_{t,i\to g}{I}_{g,t}/N}\\ {}{E}_{i,t+1}-{E}_{i,t}={S}_{i,t}{\displaystyle \sum_g^G{\beta}_{t,i\to g}{I}_{g,t}/N}-\kappa {E}_{i,t}\\ {}{I}_{i,t+1}-{I}_{i,t}=\kappa {E}_{i,t}-\gamma {I}_{i,t}\\ {}{R}_{i,t+1}-{R}_{i,t}=\gamma {I}_{i,t}.\end{array} $$

The model in (5) captures heterogeneous mixing within the population model. The group-specific transmission function (*β*_*t*,*i* → *g*_) is the same as in the homogenous case, except $\overline{C}$ and Δ_*t*_ are replaced by ${\overline{C}}_{i\to g}$ and Δ_*t*,*i* → *g*_, respectively. The parameter ${\overline{\mathrm{C}}}_{\mathrm{i}}{{}_{\to}}_{\mathrm{g}}$ reflects the average number of contacts that members of group i experience with members of group g, and *Δ*_t,i → g_ is the percent deviation from that average at time t.

C. Derivation of the log-likelihood function

We assumed that the observed number of confirmed new infections on any given day, ${I}_t^c$, follows a Poisson process with a mean arrival rate *λ*_*t*_(*ρ*):

8 $$ \Pr \left({I}_t^c\Big|{\lambda}_t\left(\rho \right)\right)=\frac{\lambda_t\left(\rho \right)}{ \exp \left({\lambda}_t\left(\rho \right)\right){I}_t^t!}. $$

The likelihood function for all observations from *t* = 1, …, *T* is given by the product:

9 $$ L={\displaystyle \prod_{t=1}^T\left(\frac{\lambda_t\left(\rho \right)}{ \exp \left({\lambda}_t\left(\rho \right)\right){I}_t^t!}\right)}. $$

Taking the log of this expression provides the log-likelihood function:

10 $$ L={\displaystyle \sum_{t=1}^T\left({I}_t^c \ln \left[{\lambda}_t\left(\rho \right)\right]-{\lambda}_t\left(\rho \right)- ln\left({I}_t^c!\right)\right)} $$

Finally, to connect the likelihood model with the SEIR transmission model, we assume that the mean Poisson arrival rate of newly confirmed cases is given by the number of new observed infections predicted by the disease model, *λ*_*t*_(*ρ*) = *ϕ*_*t*_*κE*_*t*_.

D. Cox non-nested test with a parametric bootstrap

Under a given null model (e.g. either FE or DB), each bootstrapped sample of the data (new infections) was generated by simulating draws from the Poisson process governing arrivals of new infections based on the fitted estimates of the mean arrival rate for new infections, *λ*_*t*_ ∀ *t* = 1, …, 50. This process was repeated to create M = 500 bootstrapped samples. The likelihood estimates from each of the bootstrapped samples were used to construct the following p-value [40] for the test of a given alternative model (a) against the null (0):

11 $$ p\hbox{-} \mathrm{value}=\frac{numb\left[{L}_0\left({\widehat{\theta}}_{0m},{I}_m^{obs}\right)-{L}_a\left({\widehat{\theta}}_{am},{I}_m^{obs}\right)\le {L}_{0a},\ \forall\ m=1,\dots, M\ \right] + 1\ }{M+1}, $$

where ${I}_m^{obs}$ is the bootstrapped data sample for each iteration *m* = 1, …, *M*; ${\widehat{\theta}}_{jm}$ represents the ML estimates for model *j* ∈ {*FE*, *DB*} given sample m; L_j_ is the maximum log-likelihood for the model j; ${\mathrm{L}}_{0\mathrm{a}}={\mathrm{L}}_0\left({\widehat{\uptheta}}_0\right)-{\mathrm{L}}_{\mathrm{a}}\left({\widehat{\uptheta}}_{\mathrm{a}}\right)$ is the difference between the maximum log-likelihood estimates under H_0_ and H_a_ given the original data; and numb counts the number of times the condition is true for each of M iterations. A small sample correction is implemented by adding a 1 to the numerator and denominator. Because the FE and DB models are nonnested, selection of a unique null model is not feasible. Instead, the Cox test is conducted twice, with each of the models serving as the null in turn.

E. Likelihood profiles

In Figures 6, 7, 8 we present log-likelihood profiles underlying the maximum likelihood estimates in Table 2. In each case the log-likelihood value excludes the additive constant term which is not a function of the parameters to be estimated (i.e. the final term in Equation (10)). For each profile the maximum likelihood estimates from Table 2 are indicated with a triangle.

## Endnotes

^a^Towers and Chowell [17] allow the number of contacts experienced on weekends and weekdays to differ but these levels are taken from the literature and are otherwise constant. They also allow the transmission rate to vary over time according to a first order harmonic process to capture seasonality over a large portion of the year. We do not explore this structure since our period of interest is two months long.

^b^Central Mexico includes the Federal District (Mexico City) and states of Guerrero, Hidalgo, Jalisco, Mexico (includes greater Mexico City), Puebla, San Luis Potosi, and Tlaxcala.

^c^This assumption is difficult to test for Mexico. However, data from the American Time Use Survey (http://www.bls.gov/tus/) suggest that Americans do watch more television as they spend more time in the house, though the relationship may be nonlinear [37].

^d^The data are collected and stored by the regional division IBOPE AGP Mexico (http://www.agbnielsen.net/whereweare/whereweare.asp).
