# Erratum To: Accounting for Behavioral Responses during a Flu Epidemic Using Home Television Viewing

**DOI:** 10.1186/s12879-016-1795-5

**Published:** 2016-09-05

**Authors:** Michael Springborn, Gerardo Chowell, Matthew MacLachlan, Eli P. Fenichel

**Affiliations:** 1 Department of Environmental Science & Policy, University of California, 2104 Wickson Hall, One Shields Ave., Davis, CA 95616 USA; 2 School of Public Health, Georgia State University, P.O. Box 3965, Atlanta, GA 30302-3965 USA; 3 Division of International Epidemiology and Population Studies, Fogarty International Center, National Institutes of Health, 31 Center Dr, MSC 2220, Bethesda, 20892-2220 Maryland USA; 4 Mathematical, Computational & Modeling Sciences Center, School of Human Evolution and Social Change, Arizona State University, 900 S. Cady Mall, Tempe, AZ 85287-2402 USA; 5 Department of Agricultural & Resource Economics, University of California, 2116 Social Sciences & Humanities, One Shields Ave., Davis, CA 95616 USA; 6 Yale School of Forestry and Environmental Studies, 195 Prospect St., New Haven, CT 06511 USA

## Erratum

After the publication of this work [1], we became aware of errors in the reported results in Table 1. A corrected version of this table appears below. The main error involved reporting results for the low SEL class in the high SEL class row, and vice versa. Other small errors in reported *p*-values in the final column of the table have also been corrected.

**Table 1:** Summary statistics for daily percentage deviation from the long-run mean *ATV* (Δ_*t*_) for various demographic groups

group		statistics for Δ_*t*_ within the intervention period (*τ*)			
		range	mean	mean = 0 (*p*-value)	equal means within class (*p* - value)
*aggregate*		[-1.4 %, 22.6 %]	13.6 %	<0.001	<0.001
age class	*children*	[-4.7 %, 46.2 %]	23.7 %	<0.001	
	*adults*	[-6.5 %, 21.8 %]	8.9 %	<0.001	
SEL class	*low*	[-3.5 %, 21.0 %]	11.3 %	<0.001	low-med: 0. 06
	*medium*	[0.4 %, 32.1 %]	15.3 %	<0.001	med-high: 0.40
	*high*	[-0.4 %, 31.7 %]	17.5 %	<0.001	low-high: <0.01
time of day	*daytime*	[-3.7 %, 30.7 %]	18.4 %	<0.001	<0.001
	*nighttime*	[-4.1 %, 17.0 %]	9.6 %	<0.001	

The only implication of correcting these errors for the results discussed in the paper is to strengthen rejection of one of the null hypotheses tested. In the original text we observed that, “During the intervention period, on average the high SEL group shows a response that is over 50 % greater than that of the low SEL group.” Furthermore we remarked that, “This difference is significant at the 5 % level.” This difference is in fact significant at the 1 % level. We regret the error.
